# Supplementary figures and images for: Using GIS to examine biogeographic and macroevolutionary patterns in some late Paleozoic cephalopods from the North American Midcontinent Sea
Source: PeerJ. 2019 May 13;7:e6910. doi: 10.7717/peerj.6910 (PMC6521810; doi:10.7717/peerj.6910)

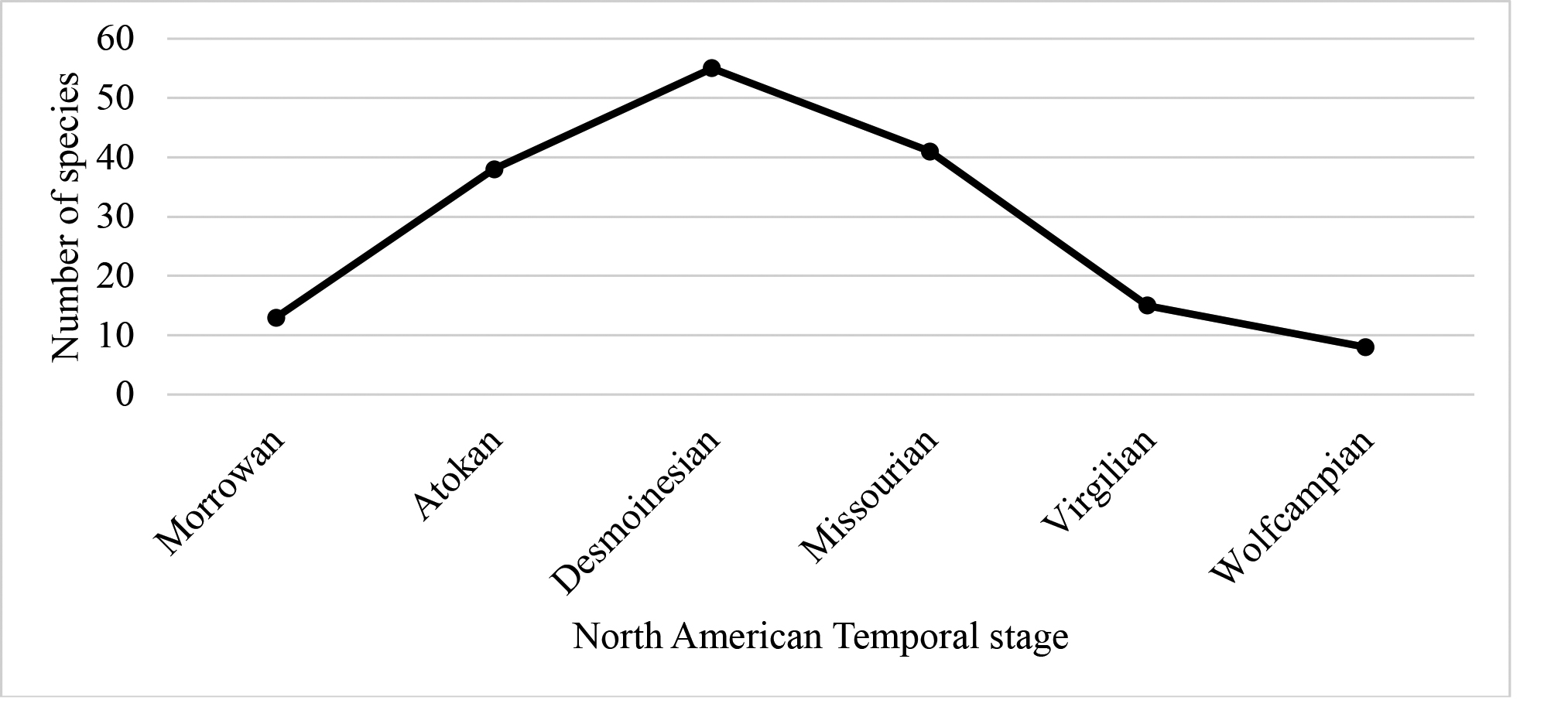

Supplement: Figure S1 — Time represented by North American temporal stage. [file peerj-07-6910-s001.jpg]

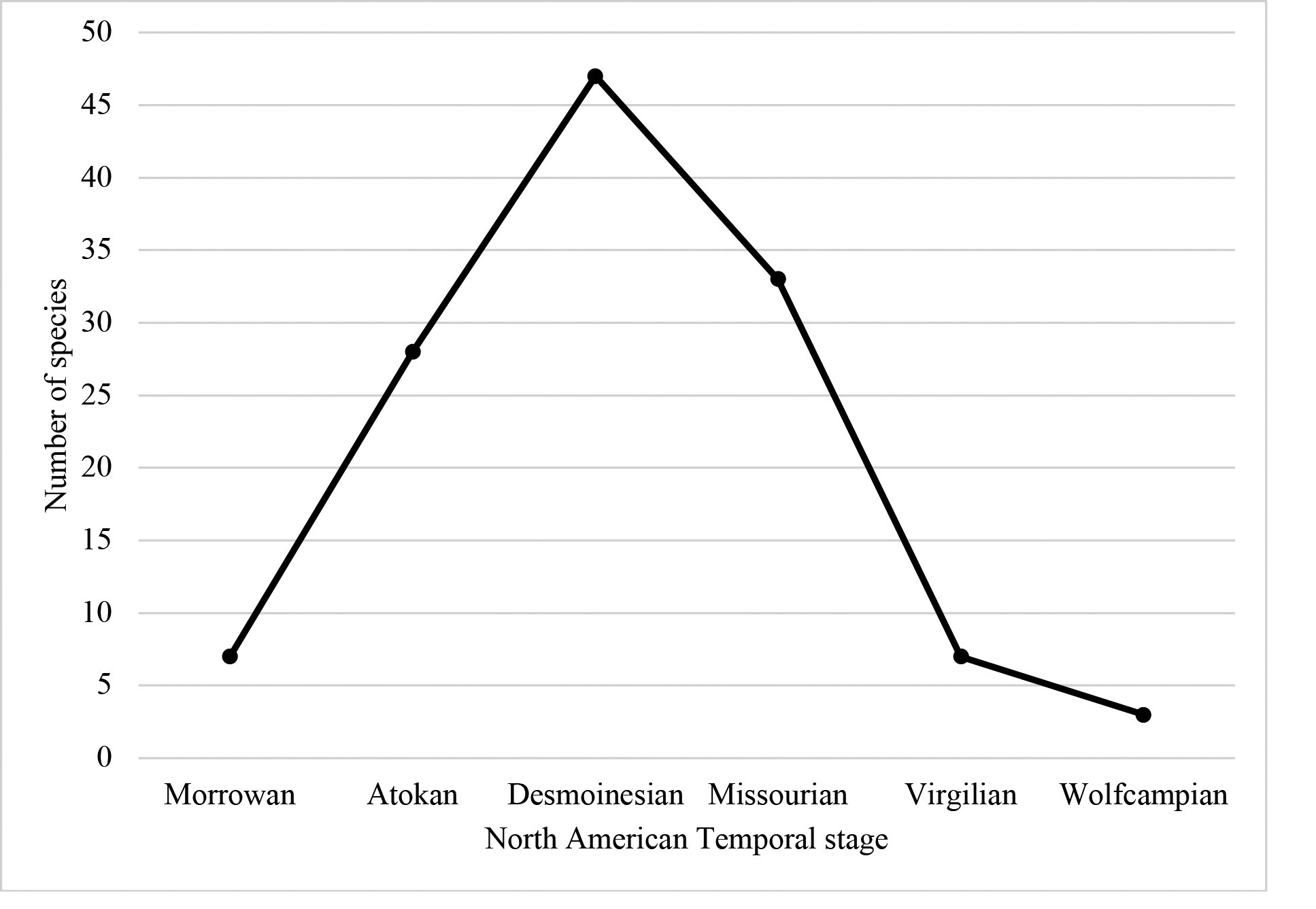

Supplement: Figure S2 — Time represented by North American temporal stage. [file peerj-07-6910-s002.jpg]

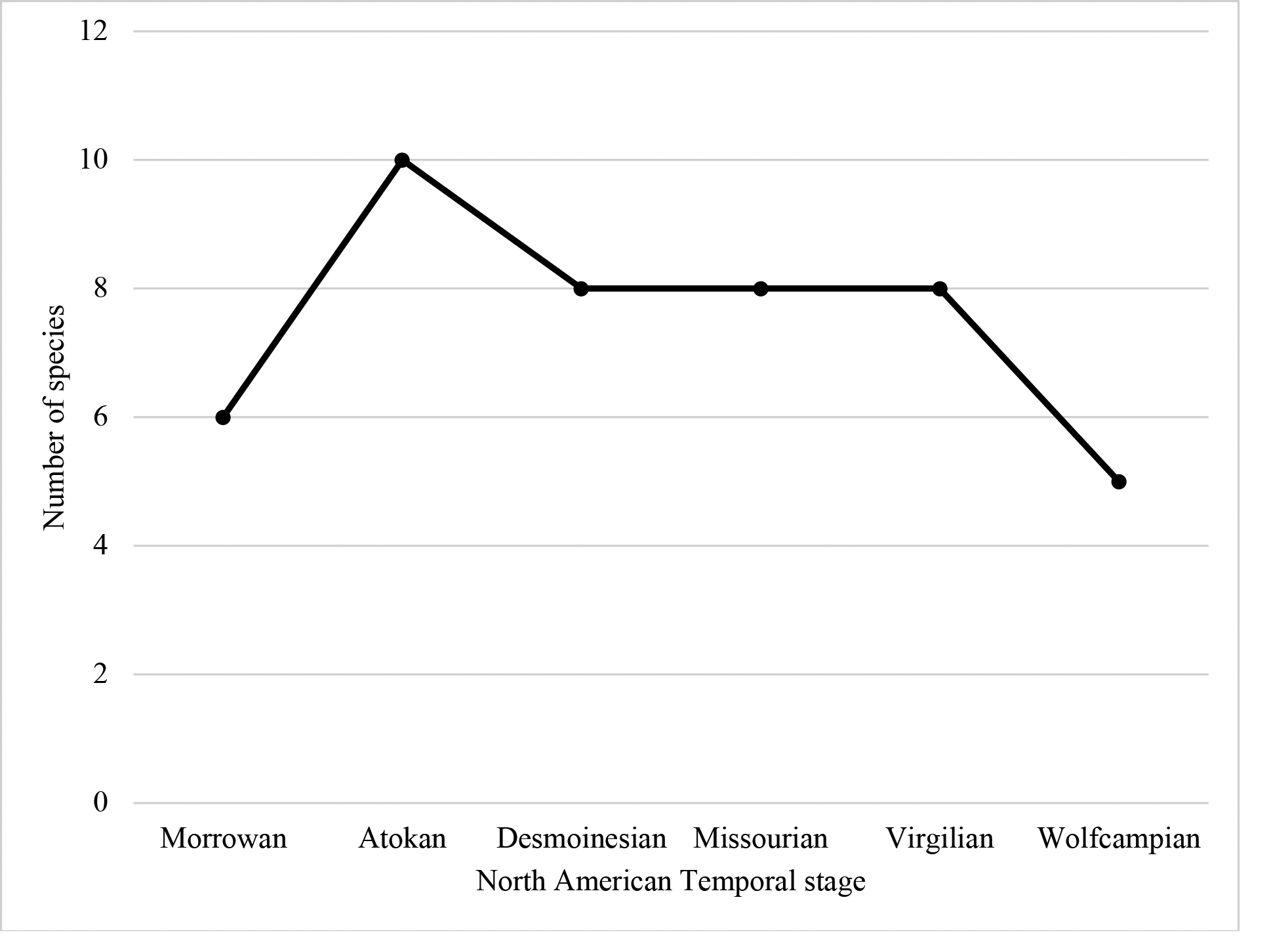

Supplement: Figure S3 — Time represented by North American temporal stage. [file peerj-07-6910-s003.jpg]
