# Supplementary material for: Using GIS to examine biogeographic and macroevolutionary patterns in some late Paleozoic cephalopods from the North American Midcontinent Sea
Source: PeerJ. 2019 May 13;7:e6910. doi: 10.7717/peerj.6910 (PMC6521810; doi:10.7717/peerj.6910)
Supplement: Table S1 [file peerj-07-6910-s004.docx]

**Supplemental Table S1:**

**List of cephalopod species considered and museum specimens used in range reconstructions, arranged alphabetically by genus and then species for Ammonoidea and Nautiloidea.**

Ammonoidea

*Gonioloboceras bridgeportense* Plummer & Scott, 1937 – YPM 15930.

*G. goniolobum* (Meek, 1877) – KUMIP 50268, 50494-50496, 50587, 50589, 65780, 65781, 150984, 151231, 151232, 151234, 151236, 151237, 151238, 151239, 151240; UI 001054, 1069, 8884B, 008884C, 010443, 10735, 13813, 51484; YPM IP-147525, 376326, 376327.

*G. gracellenae* Miller and Cline, 1934 – UI 631, 632, 632A; YPM 15302.

*G. welleri* Smith, 1903 – KUMIP 50586, 50588, 290330; UI 13808; YPM 15305, 15060, 15062, 114193, 229940.

*Gonioloboceratoides eliasi* Miller & Owen, 1939 – UI 13525-13530.

*Megapronorites baconi* (Miller, Youngquist, & Nielsen, 1952) – KUMIP 51218; UI 13945.

*Mescalites discoidalis* Böse, 1920 – YPM 13081, 13083, 14022-14058.

*Parashumardites senex* (Miller & Cline, 1934) – KUMIP 51338, 51339.

*Phaneroceras compressum* (Hyatt, 1891) – KUMIP 50375, 50365, 50368, 50420, 50431, 50438, 50457, 50367, 51101, 51116, 51126, 51127, 51133, 51147, 51168, 51169, 51197, 51211, 51217, 51234, 51244; UI 9775, 13991,17062, 61769, 61770.

*P. kesslerense* (Mather, 1915) - KUMIP 50354, 50361, 50362, 50366, 50370, 50391, 50399, 50413, 50415, 50416, 50434, 50437, 50447, 50448, 50449, 50450, 50451, 50455, 50459, 50460, 51102, 51106, 51115, 51117, 51119, 51121, 51125, 51128, 51136, 51138, 51239, 51150, 51156, 51157, 51158, 51161, 51165, 51170, 51175, 51176, 51180, 51182, 51185, 51186, 51187, 51189, 51207, 51221, 51224, 51246, 51247, 51249, 65631, 65632, 65633, 65634; UI 9741, 9743-9745, 9747-9749; YPM 10160.

*Pronorites pseudotimorensis* Miller, 1930 – YPM 12931, 12931.B, 12931.C, 147380; UI 11641. (Note has been treated as *Metapronorites*, D. Korn, pers. comm., 2018.)

*Properrinites boesei* Plummer and Scott, 1937 – UI 10616, 10618, 10620, 10621, 13607A, 13607B, 37418-37420; YPM 15964.

*P. cumminsi* (White, 1889) – YPM 15967, 25710-25713, 31227-31241; UI 5983, 52582.

*P. plummeri* Elias, 1938 – KUMIP 59718.

*Pseudoparalegoceras brazoense* Plummer & Scott, 1937 – KUMIP 50374, 50376-50378, 50394, 50412, 50421, 51103, 51104, 51113, 51135, 51164, 51179, 51188, 51208, 51210, 51213, 51230, 51252; UI 1432, 9814, 13992.

*Pseudopronorites arkansiensis* (Smith, 1896) – UI 11701, 13942-13944.

*P. kansasensis* (Newell, 1936) – KUMIP 58810.

*Schistoceras hildrethi* (Morton, 1836) - KUMIP 50272, 50493, 50653, 50954, 51194, 150988, 262460, 262461, 262484; UI 1060, 1897, 3114, 3115, 10442, 12543, 13984, 17028, 17029, 48921, 61837, 61838; YPM I114226, 147469, 376325. (Note has been treated as *Paraschistoceras*, D. Korn, pers. comm., 2018.)

*S. missouriense* (Miller & Faber, 1892) - KUMIP 32273, 51337, 58844, 65775, 151244-151249, 151871; UI 1059, 1434, 1434, 5967A, 13983, 14000, 17030, 34793, 61818 61820, 61830; YPM 12936, 12936.A, 12936.B, 12940, 75586, 7979, 114228, 114229, 229227, 229252.

*S. unicum* Miller & Owen, 1937 - KUMIP 50192, 51268, 51269, 151339-151341, 151872; UI 1433, 51480, 61827. (Note has been treated as *Eoschistoceras*, D. Korn, pers. comm., 2018.)

*Shumardites cuyleri* Plummer & Scott, 1937 – KUMIP 65823; UI 1071, 1901, 1902, 51140; YPM 15301.

*S. simondsi* Smith, 1903 – UI 38740, 38742, 53109.

*Vidrioceras uddeni* (Böse, 1919) – YPM 16812.

Nautiloidea

*Brachycycloceras bransoni* (Miller & Owen, 1934) – UI 4269, 13341-13349.

*B. crebricinctum* (Girty, 1911) – KU 53207-53211; UI 609, 4264A, 10790, 10795, 10796, 11041, 33052, 33087A; YPM 10087, 15053.

*B. curtum* (Meek & Worthen, 1860) – UI 3116, 3117, 4272, 35799; YPM 15054.

*B. longulum* Miller & Owen, 1934 – UI 13378-13382, 13384, 13385.

*B. normale* Miller, Dunbar, & Condra, 1933 – KUMIP 38566, 38765, 212024; UI 4264, 10796A, 10801, 10802, 10853, 10854, 13383, 33087, 35784, 35796; YPM 13976, 13977, 15051, 15052, 046520, 584115.

*Domatoceras bradyi* Miller & Unklesbay, 1942 – KUMIP 288716; YPM 228683.

*D. kleihegei* Miller, Lane, & Unklesbay, 1947 – KUMIP 32091, 32342, 32343, 38645, 50986, 282369.

*D. moorei* Miller, Dunbar, & Condra, 1933 – KUMIP 40872, 49570, 50952, 282318-282321; UI 1408, 6997, 51365, 51473; YPM 517369.

*D. sculptile* (Girty, 1911) – UI 6186, 13431.

*D. umbilicatum* Hyatt, 1893 – KUMIP 38619, 38621, 38624, 38653, 38654, 38674, 38675, 38677, 38678, 38681, 38685, 38686, 38689, 38690, 38692, 38832, 38833, 40882, 40883, 50944, 282307, 286896, 288739, 288786, 288896, 288897, 288898, 288899; YPM 205182.

*D. williamsi* Miller & Owen, 1934 – KUMIP 38618, 38676, 65643, 65644, 288740, 289320, 289429-289431; UI 13417, 013419, 13420, 13422, 13424, 13426, 13428, 13429, 13430-13432, 013434; YPM 205196.

*Ephippioceras ferratum* (Cox, 1857) – KUMIP 32021, 32028, 32035, 38640, 38670, 38730, 38751, 38752, 38797, 38800, 38810, 49937, 49938, 50052, 50082, 50930, 50963, 65641, 65642; UI 3128, 13388-13392, 13437, 33091; YPM 205173, 229145.

*Euloxoceras greenei* Miller, Dunbar, & Condra, 1933 – KUMIP 38571-38579, 38758, 288857, 288858; YPM 13964, 13964A, 13964B, 13965, 13965A, 13970-13974, 376171.

*Hebetorthoceras unicamera* (Smith, 1938) – KUMIP 500534, 500535, 500543, 500551-500553; YPM 228656-228658, 228662, 416223, 416224, 416226.

*Knightoceras abundum* Miller, Lane, & Unklesbay, 1947 – KUMIP 32080-32084, 38684, 40871, 40877, 40928.

*K. missouriense* Miller & Owen, 1934 – UI 13397.

*Liroceras liratum* (Girty, 1912) – KUMIP 38641, 38665, 38719, 38720, 288650, 288651, 288671, 288672, 288692, 289400-289402, 289680; UI 13939A, 13939B, 13940, 51461; YPM 229265.

*L. milleri* Newell, 1936 – KUMIP 32002-32004, 32011-32014, 32018-32020, 32361, 32362-32365, 32381, 32382, 38719, 38720, 38769, 51461, 61578, 65638, 65639, 151906, 151907, 282310, 288651, 288692, 288771, 289680; UI 51458, 51461.

*Metacoceras angulatum* Sayre, 1930 – KUMIP 32017, 32039, 38352, 38588, 38629, 38733, 38738, 38820, 40876, 40921, 40926, 50946, 282314-282316, 282379, 288706, 288707, 288709; YPM 13992, 15056.

*M. bituberculatum* Miller & Youngquist, 1949 – UI 13663.

*M. bowmani* Miller & Breed, 1964 – KUMIP 50924, 289659.

*M. cheneyi* Miller & Youngquist, 1947 – KUMIP 38555, 50966, 151882, 151883, 282322; UI 13443; YPM 174015.

*M. cornutum* Girty, 1911 – UI 3118A-3123, 8173, 13636, 13637; YPM 376050-376062, 376071, 376072, 376150.

*M. dubium* Hyatt, 1891 – KUMIP 38632, 38633, 38717, 50027, 151887-151889, 151894, 289656; YPM 229106, 229114.

*M. inconspicuum* Hyatt, 1891 – YPM 13993.

*M. jacksonense* Miller, Lane, & Unklesbay, 1947 – KUMIP 32092, 38631, 38637, 38657, 38658, 38687, 38793, 49565.

*M. knightii* Miller & Thompson, 1936 – KUMIP 32092, 38631, 38637, 38657, 38658, 38687, 38793, 49565.

*M. mutabile* Miller, Lane, & Unklesbay, 1947 – KUMIP 32032, 32036, 32040, 32070-32072, 38551, 38552, 38589-38591, 151881, 288784; UI 13399, 13402, 13403, 13443.

*M. nodosum* Miller, Dunbar, & Condra 1933 – KUMIP 38553, 38661, 40885, 50945, 151884-151886, 282384, 288831, 289439; YPM 205240.

*M. perelegans* Girty, 1915 – UI 3124-3127.

*M. sublaeve* Miller, Dunbar, & Condra, 1933 – YPM 13995, 13996A-D.

*M. sulciferum* Miller & Thomas, 1936 – KUMIP 66751; YPM 205243.

*Millkoninckioceras elaisi* (Newell, 1936) – KUMIP 32006, 40881.

*M. jewetti* (Newell, 1936) – KUMIP 38834.

*M. wyandottense* (Newell, 1936) – KUMIP 32009.

*Mooreoceras bakeri* Miller, Dunbar, & Condra, 1933 – KUMIP 32001, 32041; YPM 13959,13961.

*M. condrai* Newell, 1936 – KUMIP 32007, 38342, 38343, 38664, 44102; UI 13635; YPM 205180, 228704.

*M. conicum* Miller, Lane, & Unklesbay, 1947 – KUMIP 38601, 38604, 38609, 38696, 32074-32078; UI 13367.

*M. giganteum* Clifton, 1942 – KUMIP 38799.

*M. normale* Miller, Dunbar, & Condra, 1933 – KUMIP 32096, 38339-38341, 38502, 38503-38511, 38523, 38524, 38526, 38528, 38529, 38531, 38532, 38534, 38539, 38594, 38595, 38598-38600, 38603, 38605, 38608, 38613, 38646, 38749, 38750; UI 13351-13366, 13368-13375, 013376A, 013376B, 13684; YPM IP-013956, 013957, 228704, 229173.

*M. ovale* Young, 1942 - KUMIP 38527, 38530, 38536, 38540, 38544, 38606, 38611, 38627, 38628, 38714, 38764; YPM 228720.

*M. tuba* (Girty, 1911) - YPM 13958.

*M. wedingtonianum* Gordon, 1964 – KUMIP 38538, 38788.

*“Orthoceras” dunbari* Foerste, 1936 – YPM 10549, 228672.

*“O.” kansasense* Sayre, 1930 – KUMIP 32016, 32271, 58200-58204, 58798; YPM 15049, 15055, 228663, 228668, 228723, 228724, 376919, 376920.

*“O.” longissimicameratum* Miller, 1930 – YPM 9124, 12928A-G, 416225.

*“O.” occidentale* (Swallow, 1858) – KUMIP 32026,32030; YPM 15055, 228665.

*Pseudorthoceras knoxense* (McChesney, 1860) – KUMIP 288837-288841, 32023-32024, 38550, 38565, 38592, 38660, 38702, 500537, 500538, 500540, 52560, 65691-65697; UI 12482-12486, 13339; YPM 228354, 228360, 228364, 228365, 228379-228381, 228383-228387, 228393, 228414, 228416-228419, 228423, 228430, 228432, 229033-229036, 229148, 229150-229160.

*Solenochilius brammeri* Miller, Dunbar, & Condra, 1933 – UI 51366, 051367; YPM 14005, 14006.

*S. kempae* Miller & Youngquist, 1949 – KUMIP 38755-38757, 38794, 38806, 38819, 40886, 40888.

*S. kerefordensis* Miller, Dunbar, & Condra, 1933 – KUMIP 38824, 50077; YPM 14003.

*S. missouriense* Miller, Lane, & Unklesbay, 1947 – KUMIP 110830, 288587.

*S. newloni* Hyatt, 1891 – KUMIP 38808.

*S. peculiare* Miller & Owen, 1934 – UI 13435.

*S. springeri* (White & St. John, 1867) – KUMIP 50264, 288741; UI 11043, 12544.

*S. syracusensis* Miller, Dunbar, & Condra, 1933 – YPM 14004.
