# Supplementary material for: Using GIS to examine biogeographic and macroevolutionary patterns in some late Paleozoic cephalopods from the North American Midcontinent Sea
Source: PeerJ. 2019 May 13;7:e6910. doi: 10.7717/peerj.6910 (PMC6521810; doi:10.7717/peerj.6910)
Supplement: Table S2 [file peerj-07-6910-s005.docx]

**Supplemental Table S2:**

**Temporal boundaries (from Davydov, Korn, & Schmitz, 2012) used for calculations of late Paleozoic cephalopod speciation and extinction rates.**

| **Geologic Period** | **North American Geologic Stage** | **Upper Boundary (in Millions of Years)** |
| --- | --- | --- |
| Permian | Leonardian | 270 |
|  | Wolfcampian | 285 |
| Pennsylvanian | Virgilian | 299 |
|  | Missourian | 304 |
|  | Desmoinesian | 307 |
|  | Atokan | 310 |
|  | Morrowan | 312 |
| Mississippian | Chesterian | 318 |
