# Supplementary material for: Using GIS to examine biogeographic and macroevolutionary patterns in some late Paleozoic cephalopods from the North American Midcontinent Sea
Source: PeerJ. 2019 May 13;7:e6910. doi: 10.7717/peerj.6910 (PMC6521810; doi:10.7717/peerj.6910)
Supplement: Table S3 — ”0” indicates species is absent. [file peerj-07-6910-s006.docx]

**Supplemental Table S3:**

**Geographic range values through time in km^2^ by stage (youngest to oldest from left to right) for the species considered in the analysis. “0” indicates species is absent.**

| Genus | species | Wolfcampian | Virgilian | Missourian | Desmoinesian | Atokan | Morrowan |
| --- | --- | --- | --- | --- | --- | --- | --- |
| ***Brachycycloceras*** | ***bransoni*** | 0 | 78.539816 | 78.539816 | 0.250798 | 0 | 0 |
| ***Brachycycloceras*** | ***crebrincinctum*** | 0 | 0.523798 | 78.539816 | 1176.718476 | 0 | 0 |
| ***Brachycycloceras*** | ***curtum*** | 0 | 377.412261 | 78.539816 | 78.539816 | 0 | 0 |
| ***Brachycycloceras*** | ***longulum*** | 0 | 1065.654683 | 78.539816 | 0.833697 | 0 | 0 |
| ***Brachcycloceras*** | ***normale*** | 0 | 0.494043 | 78.539816 | 8757.000000 | 78.539816 | 0 |
| ***Domatoceras*** | ***bradyi*** | 0 | 78.539816 | 78.539816 | 0 | 0 | 0 |
| ***Domatoceras*** | ***kleihegei*** | 0 | 0 | 0.220338 | 78.539816 | 0 | 0 |
| ***Domatoceras*** | ***moorei*** | 0 | 0 | 117036.000000 | 78.539816 | 0 | 0 |
| ***Domatoceras*** | ***sculptile*** | 0 | 78.539816 | 78.539816 | 78.539816 | 0 | 0 |
| ***Domatoceras*** | ***umbilicatum*** | 78.539816 | 12010.000000 | 3440.000000 | 12489.577104 | 0 | 0 |
| ***Domatoceras*** | ***williamsi*** | 78.539816 | 78.539816 | 0.729789 | 3762.000000 | 0 | 0 |
| ***Ephippioceras*** | ***ferratum*** | 0 | 2810.000000 | 127692.000000 | 3269.000000 | 0 | 0 |
| ***Euloxoceras*** | ***greenei*** | 0 | 7594.000000 | 3827.000000 | 78.539816 | 0 | 0 |
| ***Gonioloboceras*** | ***bridgeportensis*** | 0 | 0 | 78.539816 | 0 | 0 | 0 |
| ***Mescalites*** | ***discoidalis*** | 78.539816 | 0 | 0 | 0 | 0 | 0 |
| ***Gonioloboceratoides*** | ***elaisi*** | 0 | 0 | 0 | 0.466069 | 0 | 0 |
| ***Gonioloboceras*** | ***goniolobum*** | 0 | 15174.000000 | 78.539816 | 78.539816 | 0 | 0 |
| ***Gonioloboceras*** | ***gracellenae*** | 0 | 78.539816 | 0 | 0 | 0 | 0 |
| ***Gonioloboceras*** | ***welleri*** | 0 | 115412.000000 | 78.539816 | 78.539816 | 0 | 0 |
| ***Hebetorthoceras*** | ***unicamera*** | 0 | 0 | 0 | 0.312085 | 0 | 0 |
| ***Knightoceras*** | ***abundum*** | 0 | 0 | 0.149475 | 0 | 0 | 0 |
| ***Knightoceras*** | ***missouriense*** | 0 | 0 | 0 | 78.539816 | 0 | 0 |
| ***Liroceras*** | ***liratum*** | 0 | 0 | 119598.000000 | 78.539816 | 78.539816 | 6791.691877 |
| ***Liroceras*** | ***milleri*** | 0 | 0 | 4731.000000 | 78.539816 | 78.539816 | 0 |
| ***Megapronorites*** | ***baconi*** | 0 | 0 | 0 | 0 | 78.539816 | 78.539816 |
| ***Metacoceras*** | ***angulatum*** | 78.539816 | 13702.448632 | 9458.490734 | 0.130773 | 0 | 0 |
| ***Metacoceras*** | ***bituberculatum*** | 0 | 0 | 0 | 78.539816 | 0 | 0 |
| ***Metacoceras*** | ***bowmani*** | 0 | 0 | 9231.475993 | 0 | 0 | 0 |
| ***Metacoceras*** | ***cheneyi*** | 0 | 78.539816 | 0.639810 | 78.539816 | 0 | 0 |
| ***Metacoceras*** | ***cornutum*** | 0 | 85771.000000 | 0.793486 | 670.592743 | 0 | 0 |
| ***Metacoceras*** | ***dubium*** | 5745.847979 | 78.539816 | 0.101843 | 0 | 0 | 0 |
| ***Metacoceras*** | ***inconspicuim*** | 0 | 0 | 78.539816 | 0 | 0 | 0 |
| ***Metacoceras*** | ***jacksonense*** | 0 | 78.539816 | 2214.000000 | 0 | 0 | 0 |
| ***Metacoceras*** | ***knighti*** | 0 | 0 | 0.497333 | 0 | 0 | 0 |
| ***Metacoceras*** | ***mutabile*** | 0 | 0 | 4982.000000 | 0.175450 | 0 | 0 |
| ***Metacoceras*** | ***nodosum*** | 78.539816 | 78.539816 | 4673.000000 | 78.539816 | 0 | 0 |
| ***Metacoceras*** | ***perelegans*** | 0 | 0 | 0.793486 | 0 | 0 | 0 |
| ***Metacoceras*** | ***sulciferum*** | 0 | 78.539816 | 78.539816 | 0 | 0 | 0 |
| ***Metacoceras*** | ***sublaeve*** | 78.539816 | 0 | 0 | 0 | 0 | 0 |
| ***Millkoninckioceras*** | ***elaisi*** | 0 | 0 | 1022.815493 | 0 | 0 | 0 |
| ***Millkoninckioceras*** | ***jewetti*** | 0 | 0 | 78.539816 | 0 | 0 | 0 |
| ***Millkoninckioceras*** | ***wyandottense*** | 0 | 0 | 78.539816 | 0 | 0 | 0 |
| ***Mooreoceras*** | ***bakeri*** | 0 | 4981.349200 | 4915.000000 | 0 | 0 | 0 |
| ***Mooreoceras*** | ***condrai*** | 0 | 78.539816 | 3676.000000 | 78.539816 | 78.539816 | 0 |
| ***Mooreoceras*** | ***conicum*** | 0 | 0 | 4467.000000 | 78.539816 | 0 | 0 |
| ***Mooreoceras*** | ***giganteum*** | 0 | 78.539816 | 0 | 0 | 0 | 0 |
| ***Mooreoceras*** | ***normale*** | 0 | 0 | 4979.000000 | 8202.000000 | 78.539816 | 78.539816 |
| ***Mooreoceras*** | ***ovale*** | 0 | 78.539816 | 2339.000000 | 0 | 0 | 0 |
| ***Mooreoceras*** | ***tuba*** | 0 | 0 | 78.539816 | 0 | 0 | 0 |
| ***Mooreoceras*** | ***wedingtonianum*** | 0 | 0 | 3758.113739 | 0 | 0 | 0 |
| ***“Orthoceras”*** | ***dunbari*** | 0 | 0 | 78.539816 | 78.539816 | 0 | 0 |
| ***“Orthoceras”*** | ***kansasense*** | 0 | 17195728778.000000 | 610.050914 | 78.539816 | 0 | 0 |
| ***“Orthoceras”*** | ***longissimicameratum*** | 0 | 78.539816 | 78.539816 | 78.539816 | 0 | 0 |
| ***“Orthoceras”*** | ***occidentale*** | 0 | 78.539816 | 78.539816 | 78.539816 | 0 | 0 |
| ***Parashumardites*** | ***senex*** | 0 | 78.539816 | 0 | 0 | 0 | 0 |
| ***Phaneroceras*** | ***compressum*** | 0 | 0 | 0 | 0 | 24924.000000 | 78.539816 |
| ***Phaneroceras*** | ***kesslerense*** | 0 | 0 | 0 | 0 | 0.368368 | 0 |
| ***Pseudoparalegoceras*** | ***brazoense*** | 0 | 0 | 0 | 0.505825 | 0.001144 | 0 |
| ***Pseudorthoceras*** | ***knoxense*** | 78.539816 | 71899.000000 | 7577.000000 | 64660.000000 | 30599.000000 | 0 |
| ***Pseudopronorites*** | ***arkansasensis*** | 0 | 0 | 0 | 0 | 0 | 8916.063243 |
| ***Pseudopronorites*** | ***kansasensis*** | 0 | 0 | 78.539816 | 0 | 0 | 0 |
| ***Pronorites*** | ***pseudotimorensis*** | 0 | 78.539816 | 78.539816 | 78.539816 | 78.539816 | 78.539816 |
| ***Properrinites*** | ***boesei*** | 0.559262 | 0 | 0 | 0 | 0 | 0 |
| ***Properrinites*** | ***cumminsi*** | 78.539816 | 0 | 0 | 0 | 0 | 0 |
| ***Properrinites*** | ***plummeri*** | 78.539816 | 0 | 0 | 0 | 0 | 0 |
| ***Schistoceras*** | ***hildrethi*** | 0 | 139876.000000 | 23915.000000 | 78.539816 | 78.539816 | 78.539816 |
| ***Schistoceras*** | ***missouriense*** | 78.539816 | 145678.147758 | 145678.147758 | 78.539816 | 78.539816 | 0 |
| ***Schistoceras*** | ***unicum*** | 0 | 0.275353 | 78.539816 | 78.539816 | 78.539816 | 0 |
| ***Shumardites*** | ***cuyleri*** | 0 | 0.040084 | 0 | 0 | 0 | 0 |
| ***Solenochilius*** | ***brammeri*** | 0 | 0 | 25888.015389 | 0 | 0 | 0 |
| ***Solenochilius*** | ***kempae*** | 0 | 0 | 3067.000000 | 0 | 0 | 0 |
| ***Solenochilius*** | ***kerefordensis*** | 0 | 2277.109781 | 78.539816 | 0 | 0 | 0 |
| ***Solenochilius*** | ***missouriense*** | 0 | 0 | 1321.366476 | 0 | 0 | 0 |
| ***Solenochilius*** | ***newloni*** | 0 | 78.539816 | 0 | 0 | 0 | 0 |
| ***Solenochilius*** | ***peculiare*** | 0 | 0 | 0 | 78.539816 | 0 | 0 |
| ***Shumarites*** | ***simondsi*** | 0 | 0.103678 | 0 | 0 | 0 | 0 |
| ***Solenochilius*** | ***springeri*** | 0 | 0 | 0 | 9839.217950 | 78.539816 | 78.539816 |
| ***Solenochilius*** | ***syracusense*** | 0 | 0 | 0 | 78.539816 | 0 | 0 |
| ***Vidrioceras*** | ***uddeni*** | 78.539816 | 0 | 0 | 0 | 0 | 0 |
