# Supplementary material for: Using GIS to examine biogeographic and macroevolutionary patterns in some late Paleozoic cephalopods from the North American Midcontinent Sea
Source: PeerJ. 2019 May 13;7:e6910. doi: 10.7717/peerj.6910 (PMC6521810; doi:10.7717/peerj.6910)
Supplement: Table S7 — Species richness values, species carryover from the previous stage, new species originating in the stage, No (the initial number of species), Nf (the final number of species), and duration (in Myr) also given. [file peerj-07-6910-s010.docx]

**Supplemental Table S7:**

**Speciation rates (S) per million years (Myr), extinction rates (E) per Myr, and rate of turnover (R) per Myr, for each stage for ammonoids, with species that occur in a single stage excluded, and species richness values, species carryover from the previous stage, new species originating in the stage, N_o_ (the initial number of species), N_f_ (the final number of species), and duration (in Myr) also given.**

| **Stage** | **Species Richness** | **Species Carryover** | **New Species** | **N_o_** | **N_f_** | **Duration** | **R** | **S** | **E** |
| --- | --- | --- | --- | --- | --- | --- | --- | --- | --- |
| Wolfcampian | 1 | 1 | 0 | 1 | 1 | 14 |  | 0 |  |
| Virgilian | 7 | 6 | 1 | 6 | 7 | 5 | -0.3584 | 0.0308 | 0.3892 |
| Missourian | 6 | 6 | 0 | 6 | 6 | 3 | 0 | 0 | 0 |
| Desmoinesian | 7 | 5 | 2 | 5 | 7 | 3 | 0.0608 | 0.1122 | 0.0514 |
| Atokan | 7 | 4 | 3 | 4 | 7 | 2 | 0.1116 | 0.2798 | 0.1682 |
| Morrowan | 4 | 0 | 4 | 0 | 4 | 6 |  |  |  |
