# Supplementary material for: Using GIS to examine biogeographic and macroevolutionary patterns in some late Paleozoic cephalopods from the North American Midcontinent Sea
Source: PeerJ. 2019 May 13;7:e6910. doi: 10.7717/peerj.6910 (PMC6521810; doi:10.7717/peerj.6910)
Supplement: Table S9 [file peerj-07-6910-s012.docx]

**Supplemental Table S9:**

**Percent coverage table of the range size of various species compared with available outcrop, by North American stage.**

| North American Stage:  Genus | Wolfcampian  species | Range Size of Outcrop = 1518052.708 km^2^  Range Size of Species | Percentage occupied |
| --- | --- | --- | --- |
| *Metacoceras* | *dubium* | 5745.847979 km^2^ | 0.0037850 |
| *Metacoceras* | *angulatum* | 78.539816 km^2^ | 0.0000517 |
| *Domatoceras* | *umbilicatum* | 78.539816 km^2^ | 0.0000517 |
| *Domatoceras* | *williamsi* | 78.539816 km^2^ | 0.0000517 |
| *Mescalites* | *discoidalis* | 78.539816 km^2^ | 0.0000517 |

| North American Stage:  Genus | Virgilian  species | Range Size of Outcrop = 1665938.874 km^2^  Range Size of Species | Percentage occupied |
| --- | --- | --- | --- |
| *Orthoceras* | *kansasense* | 17195700000 km^2^ | 10321.92733 |
| *Schistoceras* | *missouriense* | 145678.1478km^2^ | 0.087445074 |
| *Schistoceras* | *hildrethi* | 139876 km^2^ | 0.083962264 |
| *Gonioloboceras* | *welleri* | 115412 km^2^ | 0.069277452 |
| *Metacoceras* | *cornutum* | 85771 km^2^ | 0.051485082 |

| North American Stage:  Genus | Missourian  species | Range Size of Outcrop = 1737036.164 km^2^  Range Size of Species | Percentage occupied |
| --- | --- | --- | --- |
| *Schistoceras* | *missouriense* | 145678.1478 km^2^ | 0.083865927 |
| *Ephippioceras* | *ferratum* | 127692 km^2^ | 0.073511423 |
| *Liroceras* | *liratum* | 119598 km^2^ | 0.068851762 |
| *Domatoceras* | *moorei* | 117036 km^2^ | 0.067376836 |
| *Solenochilius* | *brammeri* | 25888.01539 km^2^ | 0.014903556 |

| North American Stage:  Genus | Desmoinesian  species | Range Size of Outcrop = 1835450.827 km^2^  Range Size of Species | Percentage occupied |
| --- | --- | --- | --- |
| *Pseudorthoceras* | *knoxense* | 64660 km^2^ | 0.035228402 |
| *Domatoceras* | *umbilicatum* | 12489.5771 km^2^ | 0.006804637 |
| *Solenochilius* | *springeri* | 9839.21795 km^2^ | 0.005360655 |
| *Brachcycloceras* | *normale* | 8757 km^2^ | 0.004771035 |
| *Mooreoceras* | *normale* | 8202 km^2^ | 0.004468657 |

| North American Stage:  Genus | Atokan  species | Range Size of Outcrop = 934292.2165 km^2^  Range Size of Species km^2^ | Percentage occupied |
| --- | --- | --- | --- |
| *Pseudorthoceras* | *knoxense* | 30599 km^2^ | 0.032750995 |
| *Phaneroceras* | *compressum* | 24924 km^2^ | 0.026676879 |
| *Brachcycloceras* | *normale* | 78.539816 km^2^ | 8.406E-05 |
| *Liroceras* | *liratum* | 78.539816 km^2^ | 8.406E-05 |
| *Liroceras* | *milleri* | 78.539816 km^2^ | 8.406E-05 |

| STAGE: | Morrowan | Range Size of Outcrop = 900649.1324 km^2^  Range Size of Species km^2^ | Percentage occupied |
| --- | --- | --- | --- |
| *Pseudopronorites* | *arkansasensis* | 8916.063243 km^2^ | 0.009899597 |
| *Liroceras* | *liratum* | 6791.691877 km^2^ | 0.007540885 |
| *Mooreoceras* | *normale* | 78.539816 km^2^ | 8.72036E-05 |
| *Phaneroceras* | *compressum* | 78.539816 km^2^ | 8.72036E-05 |
| *Megapronorites* | *baconi* | 78.539816 km^2^ | 8.72036E-05 |
